# The assessment of association between endometrioma size and other endometriosis compartments according to #ENZIAN classification

**DOI:** 10.1007/s00404-026-08354-x

**Published:** 2026-02-13

**Authors:** Elvin Piriyev, Ahmet Namazov, Islam Mahalov, Flora Huseynova, Gad Liberty, Ofer Gemer, Sven Schiermeier, Thomas Römer, Jörg Keckstein

**Affiliations:** 1 University Witten-Herdecke, Witten, Germany; 2 Department of Obstetrics and Gynecology, Academic Hospital Cologne Weyertal University of Cologne, Weyertal 76, 50931 Cologne, Germany; 3 Department of Obstetrics and Gynecology, Barzilai University Medical Center, Ashkelon, Israel; 4 Faculty of Health Sciences, Ben-Gurion University of Negev, Beer-Sheva, Israel; 5 I. M. Sechenov First State Moscow Medical University Baku Branch, Baku Health Center, Baku, Azerbaijan; 6 Endometriosis Clinic Dres. Keckstein, Villach, Austria; 7 University of Ulm, Ulm, Germany

**Keywords:** Endometriosis, #ENZIAN, Endometrioma, Size, DIE

## Abstract

**Introduction:**

Endometriomas are common in endometriosis and may coexist with deep pelvic disease. This study aimed to assess the association between endometrioma size and other endometriosis localizations using the #ENZIAN classification.

**Study design:**

This multicenter study included women who underwent laparoscopic surgery for endometriomas between 2021 and 2024, with available surgical reports defining the #ENZIAN classification. Endometriomas were categorized as O1/2 (< 7 cm) or O3 (≥ 7 cm). Other endometriosis localizations were compared between the study groups.

**Results:**

A total of 269 women underwent surgery for endometriosis involving endometriomas. Of these, 42 had #ENZIAN O3 endometriomas, with a significantly higher rate of unilateral cysts compared to women with #ENZIAN O1/2 (92.8% vs. 68.2%, p = 0.0011). After excluding bilateral cases, 194 women with unilateral endometriomas were analyzed: 39 with #ENZIAN O3 and 155 with #ENZIAN O1/2. Women with O3 lesions were significantly younger (31.5 ± 5.9 vs. 34.5 ± 6.6 years; p = 0.011). The rate of adenomyosis (#ENZIAN F-A) was significantly lower in the O3 group (41% vs. 69%, p = 0.0012), as was rectal involvement (#ENZIAN C; 7.7% vs. 23.8%, p = 0.025). The prevalence of severe pelvic wall disease (T/B ≥ 2) did not differ significantly between groups.

**Conclusion:**

Smaller endometriomas (O1/2) are associated with more extensive pelvic disease, including higher rates of adenomyosis, rectal involvement, and pelvic adhesions, whereas larger endometriomas (O3) may represent a more localized disease phenotype. These findings support the use of the #ENZIAN classification for more accurate preoperative assessment and individualized surgical planning.

## What does this study add ?

**Table:** 

Endometrioma size appears to delineate distinct phenotypes of endometriosis, with smaller cysts associated with more extensive pelvic involvement and larger cysts representing a localized disease pattern. These insights refine the characterization of endometriosis subtypes and underscore the value of the #ENZIAN classification in guiding individualized surgical strategies.	

## Introduction

Following the simplified definition, endometriosis is a benign, systemic, estrogen-dependent disease with a chronic course. It is characterized by the presence of tissue resembling endometrium located outside the uterine cavity, which induces an inflammatory-like reaction in the surrounding tissues. Its complex clinical manifestations depend on the location and extent of the lesions [1]. Although the true prevalence remains unknown, widely accepted estimates suggest that up to 10% of women of reproductive age are affected by endometriosis [2].

Three distinct forms of the disease are recognized: superficial peritoneal, deep infiltrating, and ovarian endometriosis [3]. The latter is the most commonly diagnosed type, occurring in 17–40% of symptomatic patients [4]. Ovarian endometriosis includes all lesions ≥ 5 mm infiltrating the ovarian surface [5]. Due to their frequent cystic nature, these lesions are often referred to as *endometriomas*, also known as “chocolate cysts” in reference to their dense, dark brown fluid content [6].

The origin and development of endometriomas remain subjects of debate. Existing hypotheses—derived primarily from histologic examination of tissue specimens or sonographic findings—can be reduced to two main concepts: (1) invagination of peritoneal implants from the posterior leaf of the broad ligament or the coelomic metaplasia of the ovarian surface mesothelium into the cortex, and (2) secondary colonization of functional (follicular or luteal) cysts by ectopic endometrial cells [3, 7]. These theories focus largely on the morphology of the affected ovary, explaining why some endometriotic cysts remain small while others show rapid growth. However, they do not address the potential impact of cyst size on disease course and severity.

A proper classification system is essential to understand the relationships between the various forms of endometriosis and the clinicopathological consequences of their coexistence. Since 1973, 22 classification systems have been proposed; however, even the most widely used lack comprehensiveness and reflect only certain aspects of the disease [8]. For example, the revised American Society for Reproductive Medicine (r-ASRM) classification, though favored for its simplicity, provides little detail on deep endometriosis [5, 8]. In contrast, the ENZIAN classification—evolved into the #ENZIAN system in 2019–2021 [5] to incorporate ovarian and peritoneal disease as well as pelvic adhesions—requires not only thorough surgical evaluation but also meticulous preoperative imaging, which may limit its widespread adoption.

Based on existing pathogenetic concepts suggesting that ovarian endometriomas may arise through different mechanisms—either as secondary ovarian involvement in diffuse pelvic disease or through colonization of functional ovarian cysts [9] —we hypothesized that endometrioma size may reflect distinct anatomical phenotypes of endometriosis. The rationale of this study was therefore to evaluate whether endometrioma size, as defined by the #ENZIAN classification, is associated with different distributions of endometriosis in other pelvic compartments. The null hypothesis was that endometrioma size is not associated with differences in extraovarian disease localization, whereas the alternative hypothesis was that larger and smaller endometriomas are associated with distinct patterns of pelvic disease. The correlation between endometrioma size and other endometriosis localizations according to the #ENZIAN classification has been scarcely addressed in the literature. The present study aims to investigate this relationship, with particular focus on endometriomas ≥ 7 cm as defined by the #ENZIAN scoring system.

## Methods

This multicenter, retrospective study included data from three specialized endometriosis centers: the Academic Hospital Cologne Weyertal (a certified Level III endometriosis center in Germany), Barzilai University Medical Center (an endometriosis center in Ashkelon, Israel), and Baku Health Center (an endometriosis center in Baku, Azerbaijan). The study was approved by the Institutional Review Board (Protocol No. 78/2025). A total of 269 consecutive medical records from women who underwent laparoscopic surgery for ovarian endometrioma between 2022 and 2024 were retrospectively identified at the participating centers. Women were eligible for inclusion if they (1) underwent laparoscopic surgery with symptomatic ovarian endometrioma, (2) had histological confirmation of endometrioma, and (3) had a complete operative report documenting the #ENZIAN classification. Patients with incomplete surgical documentation or missing #ENZIAN staging were excluded.

All patients underwent laparoscopy, enabling a comprehensive evaluation of peritoneal endometriosis and adhesions. Histological confirmation of endometriomas was mandatory. Endometrioma size was determined using transvaginal ultrasound. The diagnoses of adenomyosis and deep infiltrating endometriosis (DIE) were established through transvaginal ultrasound, magnetic resonance imaging (MRI), and laparoscopy. The MUSA criteria were applied for ultrasound-based assessment of adenomyosis [10].

Endometriosis staging was performed according to the #ENZIAN classification [5], with a 7 cm cut-off used to differentiate between smaller (O1/2) and larger (O3) endometriomas, in line with #ENZIAN guidelines. The coexistence of endometriomas with adhesions, peritoneal endometriosis, and DIE, as well as their extent according to the #ENZIAN classification, was analyzed in relation to endometrioma size and laterality.

### Statistical analysis

Statistical analysis was performed using Stata/MP 16.1 software (StataCorp, College Station, TX, USA). Data are presented as numbers and percentages for qualitative variables, and as mean ± standard deviation (SD), median, and range for continuous variables. Group comparisons were conducted using the Fisher´s exact test for categorical variables and the Student’s *t*-test for continuous variables. A *p*-value < 0.05 was considered statistically significant.

## Results

A total of 269 women underwent surgery for endometriosis involving endometriomas (Table 1 summarizes demographic and clinical characteristics). Forty-two women had #ENZIAN O3 endometriomas; among these, 39 were unilateral (92.8%). In contrast, among women with #ENZIAN O1/2 endometriomas (n = 227), 155 (68.2%) were unilateral—a statistically significant difference (*p* = 0.0011).

**Table 1:** Demographic and clinical characteristics of the study population

	Total = 269 N (%); mean ± SD
Age	33.89 ± 6.55
Body mass index (kg/m^2^)	23.95 ± 4.55
Previous gynecologic surgery	166 (61.7%)
Previous endometriosis surgery	72 (26.8%)
Fertility desire	199 (73.9%)
Documented infertility	56 (20.8%)
Preoperative hormonal treatment	62 (23.4%)
Surgeries for endometioma (n = 269)	
Cystectomy	226 (84.01%)
Ethanol	20 (7.43%)
Sclerotherapy	13 (4.83%)
Oophorectomy	7 (2.6%)
Laser	3 (1.12)
Ablation	
Aspiration	
Surgeries for bowel endometriosis (n = 30)	
Shaving	18 (60%)
Bowel	9 (30%)
Resection	3 (10%)
Discoid	
Other surgeries (n = 27)	
Hysterectomy	15 (32.6%)
Salpingectomy	22 (47.83%)
Myomectomy	9 (19.57%)

Because bilateral endometriomas are often associated with severe deep infiltrating endometriosis (DIE) and extensive adhesions, bilateral cases were excluded from comparative analyses to focus on unilateral disease. The analytic cohort comprised 194 women with unilateral endometriomas: 39 with #ENZIAN O3 and 155 with #ENZIAN O1/2. Table 2 compares baseline characteristics. Women with #ENZIAN O3 were younger than those with #ENZIAN O1/2 (31.54 ± 5.93 vs. 34.48 ± 6.58 years; *p* = 0.0118).

**Table 2:** Comparison of clinical and demographic characteristics between groups in women with unilateral Endometrioma

	#ENZIAN O3 (n = 39)	#ENZIAN O1,2 (n = 155)	P
Age	31.54 ± 5.93	34.48 ± 6.58	**0.0118**
BMI	23.82 ± 4.69	24 ± 4.55	0.7883
Symptoms*			
Dysmenorrhea	28 (93.3%)	129 (93.47%)	1.0000
Dysmenorrhea VAS score	7.32 ± 2.65	7.87 ± 1.95	0.2106
Dyspareunia	10 (33.3%)	74 (56.9%)	0.0686
Dyspareunia VAS score	6.29 ± 2.84	6.36 ± 2.48	0.9150
Dyschezia	7 (23.2%)	50 (36.2%)	0.2065
Dyschezia VAS score	5.86 ± 2.41	5.98 ± 2.39	0.8990

^*^data about symptoms were available in 168 women ( #ENZIAN O3 = 30; #ENZIAN O1,2 = 138)

The rate of complete Douglas pouch obliteration was comparable between groups (15.1% vs. 15.2%; *p* = 0.1924). In the study population (n = 194), the incidences of #ENZIAN F-U, F-B, F-I, and F-diaphragm involvement were 1.5%, 1.5%, 6.2%, and 5.15%, respectively; due to their low frequencies, these compartments were not included in the comparative analysis.

Because patients with O1/O2 endometriomas were older, age was evaluated as a potential confounder using multivariable logistic regression. Age was not independently associated with adenomyosis (OR 1.004, 95% CI 0.96–1.05, p = 0.88) or rectal deep infiltrating endometriosis (OR 1.003, 95% CI 0.95–1.06, p = 0.98). In contrast, small endometriomas (#ENZIAN O1/O2) were independently associated with adenomyosis (OR 3.17, 95% CI 1.52–6.17, p = 0.02) and rectal deep infiltrating endometriosis (OR 4.00, 95% CI 1.15–13.86, p = 0.029).

As shown in Table 3, lesion distribution in other #ENZIAN compartments differed between groups. Adenomyosis (#ENZIAN F-A) was significantly less frequent in women with #ENZIAN O3 (41% vs. 69%; *p* = 0.0016). Rectal involvement (#ENZIAN C) was also lower in the O3 group (7.7% vs. 23.8%; *p* = 0.0266). Regarding pelvic wall disease (T and B compartments), bilateral T-compartment involvement was significantly less frequent in the O3 group (Table 3), whereas B-compartment distribution was comparable between groups.

**Table 3:** Surgical findings and #ENZIAN localizations according to groups in women with unilateral endometrioma

	#ENZIAN O3 (n = 39)	#ENZIAN O1,2 (n = 155)	P
#ENZIAN FA	16 (41%)	107 (69%)	**0.0016**
#ENZIAN P	32 (82%)	129 (83,2%)	0.8155
#ENZIAN T			**0.0199**
Not observed	6 (15.38%)	9 (5.81%)	
Ipsilateral	25 (64.10%)	76 (49.03%)	
Contralateral	1 (2.56%)	6 (3.87%)	
Bilateral	7 (17.95%)	64 (41.29%)	
#ENZIAN A	13 (33.3%)	61 (39.35%)	0.5814
#ENZIAN B			0.3148
Not observed	22 (56.41%)	72 (46.45%)	
Ipsilateral	12 (30.77%)	38 (25.16%)	
Contralateral	0	4 (2.58%)	
Bilateral	5 (12.82%)	41 (26.45%)	
#ENZIAN C	3 (7.69%)	37 (23.8%)	**0.0266**

In multivariable logistic regression analysis, neither previous gynecologic surgery (OR 1.00, 95% CI 0.00–∞, p = 0.998) nor previous endometriosis surgery (OR 1.00, 95% CI 0.00–∞, p = 0.963) was independently associated with #ENZIAN T. In contrast, small endometriomas (#ENZIAN O1/O2) were significantly and independently associated with #ENZIAN T (OR 4.41, 95% CI 1.41–13.70, p = 0.011).

Given the importance of preoperative recognition of pelvic wall disease for surgical planning, we further examined severe lesions (T/B ≥ 2). In a subgroup analysis assessing coexisting T ≥ 2 and B ≥ 2 involvement (Table 4), no statistically significant differences were observed between groups.

**Table 4:** Comparison of severe pelvic wall findings according to the #ENZIAN classification between two groups

	#ENZIAN O3 (n = 39)	#ENZIAN O1,2 (n = 155)	P
#ENZIAN T ≥ 2 and B ≥ 2			0.6727
Not observed	26 (66.7%)	99 (63.9%	
Ipsilateral	8 (20.5%)	41 (26.5%)	
Contralateral	4 (10.3%)	9 (5.8%)	
Bilateral	1 (2.6%)	6 (3.9%)	

A post hoc power analysis for adenomyosis prevalence between groups showed a statistical power of 89.8%, which is generally considered adequate.

## Discussion

In this study, comparison of larger (#ENZIAN O3) and smaller (#ENZIAN O1/2) endometriomas revealed a distinctive pattern: women with smaller cysts tended to be older and exhibited more extensive disease, including a higher prevalence of bilateral tubo-ovarian adhesions, adenomyosis, and rectal involvement.

This finding aligns with previous reports suggesting a link between adenomyosis and deep rectal endometriosis, potentially explained by anatomical proximity and shared pathogenic mechanisms. Donnez et al. reported a high rate of co-occurrence between deep endometriotic nodules in the posterior compartment and extrinsic adenomyosis [11]. In another retrospective MRI-based study, the prevalence of deep rectal endometriosis was significantly higher in women with both anterior and posterior adenomyosis [12]. Furthermore, rectal endometriosis and adenomyosis may represent two manifestations of the same disease. Bulun et al. demonstrated that, histologically, adenomyosis and deep endometriosis share similar features, including macrophage infiltration, fibrosis, and KRAS mutations [13, 14]. While our findings are consistent with these observations, the hypothesis of a common or similar pathogenesis between adenomyosis and rectal endometriosis should be interpreted with caution, as our data do not establish causality. We have therefore revised our interpretation to better reflect the limitations of the evidence presented. On one hand, our findings align with previous reports and support the assumption of a common or similar pathogenesis for adenomyosis and rectal endometriosis [12, 15, 16]. On the other hand, based on our data, adenomyosis was significantly more prevalent in patients with #ENZIAN O1/2 endometriomas than in those with O3 lesions, suggesting a potential interaction between adenomyosis and more diffuse pelvic disease. We hypothesize that adenomyosis may contribute to a broader inflammatory and fibrotic pelvic environment, which in turn increases the likelihood of pelvic wall disease and deep infiltrating endometriosis. If this association reflects an advanced stage of the disease, the lesion mapping provided by the #ENZIAN classification enables a logical extension of this concept: such extensive pelvic involvement may facilitate secondary ovarian involvement, leading to the development of endometriomas as an extension of pelvic disease rather than as isolated ovarian lesions. Within this framework, and in line with pathogenetic concepts proposed by Nezhat, ovarian involvement arising from pelvic disease is more likely to result in smaller endometriomas, which are often accompanied by fibrosis and adhesions that limit cyst expansion. In contrast, larger endometriomas may be secondary to pre-existing functional cysts or cystic ovarian tumors of a different origin**.** [9].

Ovarian endometriosis is rarely an isolated finding and may even be considered a marker of advanced-stage disease [17–22]. Previous studies have applied arbitrary size cut-offs of 4 cm or 7 cm but found no significant differences in the prevalence of DIE among patients with endometriomas, despite a high frequency of pelvic (paraovarian) adhesions. A likely explanation is the lack of standardized lesion mapping using the #ENZIAN classification, which may have limited the precision of lesion localization and severity assessment [20, 21]. The cut-off for endometriomas in the #ENZIAN classification was established based on expert consensus, with particular attention to lesions ≥ 7 cm (O3). This grading system allows for a more precise assessment of disease extent, thereby enhancing the interpretive value of our findings. The present study further supports the use of this 7 cm threshold for defining large cysts. In our cohort, smaller cysts (O1/2) were more frequently associated with contralateral or bilateral adhesions without pronounced ipsilateral involvement, whereas large cysts (O3) were more often unilateral (92.8% vs. 68.2%; *p* = 0.0011). Additionally, O3 lesions showed significantly lower rates of adenomyosis (41% vs. 69%; *p* = 0.0016), rectal involvement (7.69% vs. 23.8%; *p* = 0.0266), and bilateral T-compartment disease (17.95% vs. 41.29%; *p* = 0.0199).

These patterns suggest that:

1. Disease in the O1/2 group is more extensive within the pelvis;
2. Adhesions in the O1/2 group likely result from peritoneal implants;
3. O3 cysts may represent a more localized disease phenotype, sharing characteristics of functional cysts, as described by Nezhat et al. as Type II endometriomas [23].

These findings are also consistent with the younger age observed in the O3 group (31.54 ± 5.93 vs. 34.48 ± 6.58 years; *p* = 0.0118). Although data on the association between patient age and endometrioma size are not uniform, there is evidence suggesting that younger women may present with larger endometriomas. A histopathological analysis reported that patients with large endometriotic cysts were on average younger than those with smaller cysts [24]. This observation can be explained by different pathogenetic mechanisms of ovarian endometrioma formation, which we described above. Functional cysts affected by endometriosis may therefore grow rapidly and reach larger sizes, particularly in younger women with active ovulatory function. In contrast, smaller endometriomas may reflect secondary ovarian involvement in longstanding pelvic disease, often limited by fibrosis and adhesions [9, 25].

In our multivariable analyses, age was not independently associated with either adenomyosis or rectal deep infiltrating endometriosis; however, smaller endometriomas (O1/O2) remained significantly associated with both conditions. This finding suggests that the observed anatomical distribution reflects a distinct disease phenotype rather than an age-driven effect alone.

Although prior surgery is a potential risk factor for periovarian adhesions, our regression analysis indicates that the association between small endometriomas and #ENZIAN T is independent of previous gynecologic or endometriosis surgery. This finding supports the concept that O1/O2 endometriomas may represent a disease phenotype associated with more diffuse pelvic involvement, rather than being explained by postoperative sequelae alone.

Ovarian endometriosis and its surgical treatment are well-recognized factors that may negatively affect fertility [26]. In our cohort, however, the incidence of documented infertility was relatively low, and a formal comparison between #ENZIAN O3 and O1/2 groups was not performed. This was primarily due to the exclusion of bilateral endometriomas from the comparative analysis, as bilateral ovarian involvement is a major determinant of reproductive prognosis and could have confounded the interpretation of size-related effects. Consequently, while both O3 and O1/2 lesions should be considered a relevant fertility concern, the present study was not designed to assess fertility outcomes or to draw conclusions regarding differential reproductive prognosis between these phenotypes.

In our multicenter cohort, endometrioma management was performed according to local practice and surgeon preference, with no standardized approach applied. While cystectomy remains widely used, there is an increasing tendency toward tissue-sparing alternatives such as laser ablation or sclerotherapy; however, no single universally accepted strategy exists across countries, and treatment should be individualized, independent of cyst size, based on disease phenotype, fertility goals, and surgical expertise [27]. As presented in the Table 1, in our cohort the surgical management of endometrioma was performed by several approaches independently to the size of endometrioma.

We also hypothesize that the development of compartments A and B is independent of the O compartment, which may explain the similar prevalence observed in both groups.

Our study further highlights the potential strengths of the #ENZIAN classification. Compared with previous staging systems, it offers a structured, coded description of both the location and extent of endometriosis—analogous to the TNM system in oncology—and establishes a common terminology and scoring framework for clinicians, surgeons, and radiologists. This standardized approach may facilitate a more consistent characterization of disease phenotypes that have been previously described but remain insufficiently understood or validated, such as endometriomas of different sizes. Through the systematic mapping of anatomical compartments and documentation of lesion size, the classification may also reflect underlying disease mechanisms. Nevertheless, while #ENZIAN may enhance clinical communication and support more informed decision-making regarding hormonal therapy, assisted reproductive technologies, and surgical management, further studies are required to determine its prognostic value and impact on clinical outcomes.

## Limitation

The exclusion of bilateral endometriomas warrants further clarification. Such cases often represent more advanced disease and can provide valuable insights in comprehensive surgical assessments. However, they were excluded from the main comparative analysis to reduce confounding and better isolate the relationship between cyst size and disease distribution. Bilateral cases are typically associated with more widespread pelvic disease and DIE, which could obscure the potential role of cyst size alone. We acknowledge that this exclusion limits the generalizability of our findings and have highlighted it as a study limitation. Moreover, important potential confounding factors—such as hormonal treatment history, parity, and body mass index (BMI)—were not included in our analysis. These variables may significantly influence endometriosis progression, lesion size, and lesion distribution, and should be addressed in future research. A further limitation is that endometrioma size was assessed at the time of surgery, and lesion growth during observation cannot be excluded. Information on initial lesion size at diagnosis was not consistently available due to the retrospective design. However, only 15 patients (3 in the O3 group and 12 in the O1/2 group, p = 1.0000) had undergone previous surgery for ovarian cysts, suggesting that prolonged observation or repeated surgical intervention was uncommon in this cohort. The relatively small number of patients with large endometriomas (#ENZIAN O3) represents a limitation and may have reduced the statistical power to detect smaller intergroup differences. However, a post hoc power analysis based on the difference in adenomyosis prevalence between groups demonstrated a statistical power of 89.8%, supporting the robustness of the primary findings. Nonetheless, results regarding less frequent compartments should be interpreted with caution. Finally, the retrospective design of this study and the absence of key postoperative outcomes—such as ovarian reserve, pain scores, and recurrence rates—limit the clinical applicability of our conclusions. Although we identified distinct anatomical associations, their value in guiding surgical decision-making or patient counselling remains speculative. Prospective studies incorporating surgical outcomes and long-term follow-up are warranted to validate and expand upon these findings.

## Data Availability

No datasets were generated or analysed during the current study.
